# 6′-Sialyllactose Enhances Exercise Performance via Increased Muscle Mass and Strength

**DOI:** 10.3390/nu16162600

**Published:** 2024-08-07

**Authors:** Eun-Jung Park, Li-La Kim, Jie-Oh Lee, Hay-Young Lee, Yong-An Kim, Hi-Roe Go

**Affiliations:** 1 GeneChem Inc., Daejeon 34025, Republic of Korea; sksgy@genechem.co.kr (E.-J.P.); lilack8899@gmail.com (L.-L.K.); 2 Department of Life Sciences, Pohang University of Science and Technology (POSTECH), Pohang 37673, Republic of Korea; jieoh@postech.ac.kr; 3 POSTECH Biotech Center, Pohang University of Science and Technology (POSTECH), Pohang 37673, Republic of Korea; hayyoung@postech.ac.kr

**Keywords:** 6′-sialyllactose, exercise performance, muscle strength, muscle mass

## Abstract

Sialyllactose (SL) is a functional human milk oligosaccharide essential for immune support, brain development, intestinal maturation, and antiviral defense. However, despite its established health benefits, the effect of SL on exercise performance and muscle mass in mice remains unknown. Here, we aimed to investigate, for the first time, the effects of 6′-SL on muscle functions. Seven-week-old male C57BL/6J mice were administered 100 mg/kg 6′-SL for 12 weeks, after which exhaustive treadmill performance was conducted. Moreover, muscle strength was examined by grip strength, and muscle phenotype characteristics such as muscle mass, muscle fiber size, and muscle protein expression were also examined. The administration of 6′-SL significantly improved exhaustive treadmill performance metrics, including distance and exhaustion time. Grip strength was also increased by 6′-SL administration. Additionally, 6′-SL increased muscle mass in both the gastrocnemius (GAS) and soleus. 6′-SL administration led to an increase in the minimum Feret’s diameter and the protein expression of total myosin heavy chain in the GAS muscle. In conclusion, 6′-SL administration in vivo led to increased running distance and time by increasing muscle mass and strength. These findings collectively indicate that 6′-SL is a potential agent for improving muscle health and exercise performance.

## 1. Introduction 

Exercise performance is influenced by various factors, including muscle fiber composition, metabolic efficiency, muscle mass, muscle strength, and neuromuscular coordination [1,2,3]. Many studies corroborate that increased muscle strength and mass facilitate the execution of common sports skills such as jumping, sprinting, and changing direction [4,5]. Strength training and dietary supplements are established strategies for increasing muscle mass, strength, and overall health [6]. Athletes and fitness enthusiasts commonly use various dietary supplements [7,8], such as creatine, protein powders, β-hydroxy β-methylbutyrate (β-HMB), chromium, vanadyl sulfate, boron, and leucine, all known to enhance muscle mass and strength [9,10]. However, long-term or high-dose usage of these supplements may lead to potential side effects, such as gastrointestinal disturbances and drug interactions [11]. Moreover, concerns about lack of efficacy, financial expense, lack of quality control, potential to produce significant toxicity, and little attention to dietary supplement regulation in public health have been raised [11,12]. Despite widespread consumption, thousands of dietary supplements have been produced and sold without strict regulation. Unlike food and drugs, dietary supplements do not have to be registered or approved by the FDA before they can be manufactured or sold [12].

Although creatine supplementation is particularly popular for muscle growth, its effectiveness and safety remain debated [13]. Therefore, safe and accessible alternatives to address these concerns are imperative [4].

Human milk oligosaccharides (HMOs), abundant in breast milk, play a pivotal role in several bioactive functions and significantly contribute to the nutritional value of breast milk [14]. Over 100 different HMOs, including sialylated and fucosylated oligosaccharides, have been identified to date [14]. Sialyllactose (SL) is the most abundant sialylated oligosaccharide, characterized by the linkage of *N*-acetylneuraminic acid to the galactosyl subunit of lactose. SL confers various health benefits and plays crucial roles in several physiological processes, such as gastrointestinal microbiota development, gut maturation, brain and cognitive development, and the enhancement of innate immunity as a decoy receptor for viruses, potential pathogens, and bacteria [15]. The predominant forms of SL are 3′-SL (with *N*-acetylneuraminic acid connected to the 3′ position of lactose) and 6′-SL (with *N*-acetylneuraminic acid connected to the 6′ position of lactose), of which 6′-SL exerts several therapeutic effects on diseases such as necrotizing enterocolitis, neuritogenesis, and benign prostatic hyperplasia [16,17,18].

In relation to muscle health, 6′-SL has also been demonstrated to ameliorate myopathic phenotypes, such as muscle weight and locomotor activity, in symptomatic bifunctional UDP-*N*-acetylglucosamine 2-epimerase/*N*-acetylmannosamine kinase (GNE) myopathy [19]. A recent study further demonstrated that 6′-SL could increase limb muscle power in patients with GNE myopathy [20]. Although ameliorative effects of 6′-SL on myopathy have been reported, the efficacy of 6′-SL on muscle function under normal conditions has not been evaluated. Hence, in the present study, we aimed to investigate the effects of 6′-SL on exercise performance capacity and muscle phenotype in normal young mice. This study will provide evidence for the use of 6′-SL as a supplement for athletes and sports enthusiasts.

## 2. Materials and Methods

### 2.1. Animals and Treatments

Four-week-old male C57BL/6J mice, weighing 19–22 g, were obtained from Daehan Biolink (Seoul, Republic of Korea) and housed in a controlled environment (22–23 °C, 12/12 h light/dark cycle), with ad libitum access to water and a normal diet. This study was conducted at Pohang Technopark Biotechnology Center in accordance with the guidelines of the Pohang Technopark Animal Ethics Committee (ABCC 2022009, Pohang, Republic of Korea). After a 3-week acclimatization period, the mice (*n* = 20) were randomly divided by the investigators into two groups based on body weight. Group 1 (*n* = 10) was orally administered with water, whereas Group 2 (*n* = 10) was administered 100 mg/kg 6′-SL in water. We selected the dose by referring to other SL papers that showed efficacy. Both groups were treated once a day, 5 days per week, for 12 weeks. Animals’ health was monitored once daily, 5 days per week, through animal activity, panting, and fur condition. The following analyses were assessed: muscle weight measurement; exhaustive treadmill exercise test; forelimb grip strength test; dual-energy X-ray absorptiometry measurement; muscle fiber size measurement; and muscle protein expression. The 6′-SL used in this study was produced via enzyme synthesis by GeneChem, Inc. (Daejeon, Republic of Korea). All experimental procedures were approved by the Institutional Ethics Committee for the Care and Use of Animals.

### 2.2. Sample Collection

After 12 weeks of 6′-SL administration, mice were humanely euthanized, and gastrocnemius (GAS) and soleus (SOL) muscles were harvested. GAS and SOL muscle tissues were weighed, and GAS muscle tissues were either frozen in liquid nitrogen for protein extraction or fixed in 10% formalin for histological staining. Immediately after necropsy, analysis was performed.

### 2.3. Exercise Function Measurement

After 10 weeks of 6′-SL administration, an exhaustive treadmill exercise test was conducted. The test began at a speed of 10 m/min on a flat surface (0% slope) for 3 min. Subsequently, the speed was gradually increased to 20 m/min and maintained until the mice reached exhaustion, which was defined as the inability to continue running for 10 s [21]. A forelimb grip strength test was conducted at 3- and 10-weeks post-6′-SL administration using a maximal voluntary force testing system (BIO-G53; BIOSEB, Pinellas Park, FL, USA) [22].

### 2.4. Dual-Energy X-ray Absorptiometry Measurement

After 12 weeks of 6′-SL administration, fat mass, bone mineral content (BMC), bone mineral density (BMD), bone area, and bone volume were measured using dual-energy X-ray absorptiometry (DEXA; InAlyzer; MEDIKORS, Seongnam-si, Republic of Korea).

### 2.5. Histological Tissue Staining

Formalin-fixed GAS muscles were embedded in paraffin and cut into 4 μm thick slices, stained with hematoxylin and eosin (H&E), and imaged using a Digital Fluorescence Slide Scanner (Axio Scan Z1, Carl Zeiss Microscopy GmbH, Jena, Germany). The minimum Feret’s diameter of muscle fibers and their percentage distribution were measured using ImageJ 1.53t [23].

### 2.6. Western Blotting

Total protein was isolated from GAS muscles using the T-PER™ Tissue Protein Extraction Reagent (78510; Thermo Fisher, Rockford, IL, USA). Protein concentration was determined using the Quick Start™ Bradford Protein Assay (5000202; Bio-Rad Laboratories, Hercules, CA, USA). Equal amounts of protein were loaded onto 4–20% Mini-PROTEAN^®^ TGX™ Precast Protein Gels (4561096; Bio-Rad Laboratories, Hercules, CA, USA). Subsequently, the resolved proteins were transferred onto a polyvinylidene difluoride membrane using the Trans-Blot Turbo Transfer System (1704156; Bio-Rad Laboratories, Hercules, CA, USA) and subjected to immunoblotting using anti-total myosin heavy chain (MHC) (sc-376157; Santa Cruz Biotechnology (SCBT), Dallas, Texas, USA) and anti-α-tubulin (sc-5286; Santa Cruz Biotechnology (SCBT)) overnight at 4 °C. Next, the membranes were incubated with the secondary antibodies (LF-SA8001; Abfrontier, Seoul, Republic of Korea) at room temperature for 1 h. After three washes with Tris-buffered saline containing 0.1% Tween, protein bands were detected using an enhanced chemiluminescence reagent (1705061; Bio-Rad Laboratories, Hercules, CA, USA).

### 2.7. Statistical Analysis

Data are presented as mean ± standard deviation. The sample size for each experiment was selected to calculate significant differences. Animals were excluded if they died prematurely, or tissue samples were lost during autopsy. The number of animals was adjusted between the control and 6′-SL treatment groups (muscle weight measurement, exhaustive treadmill exercise test, forelimb grip strength test, and dual-energy X-ray absorptiometry measurement, *n* = 8; muscle fiber size measurement and muscle protein expression, *n* = 3). All statistical analyses were performed using GraphPad Prism 10.1.0 (GraphPad Software, Inc., San Diego, CA, USA). Significant differences were determined using a two-tailed unpaired *t*-test followed by Dunnett’s multiple comparison test. Results with *p* < 0.05 were considered significant.

## 3. Results

### 3.1. 6′-SL Enhances Muscle Function

**Figure 1:**
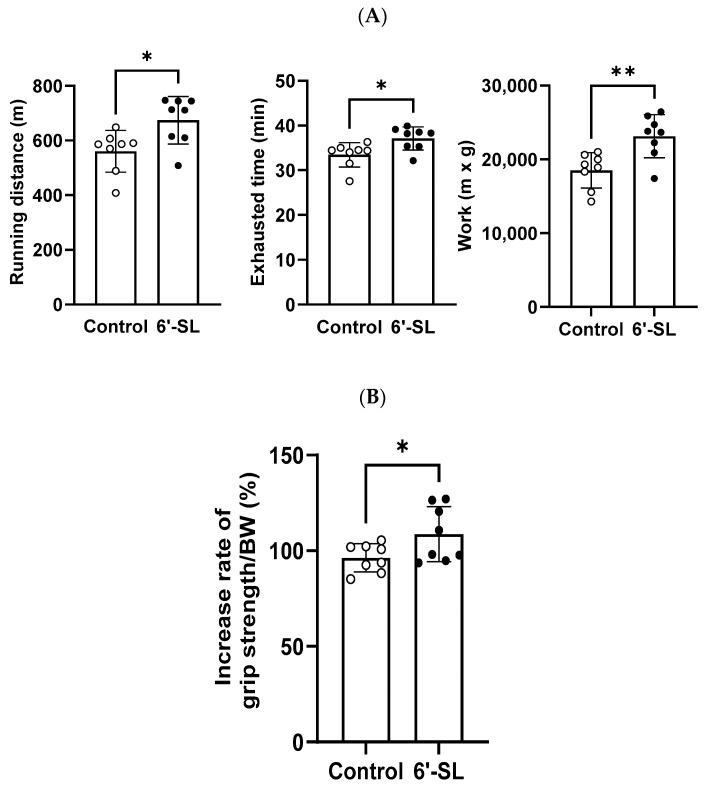
Effect of 6′-SL on exercise performance and muscle strength. (**A**) Total running distance, exhaustion time, and work during exhaustive exercise were measured at 10 weeks. Work was calculated as running distance (m) × body weight (g). (**B**) Rate of increase in grip strength/body weight between weeks 3 and 10. Data are presented as mean ± standard deviation values for each group (*n* = 8). White dots mean control groups and black dots mean 6′-SL groups. A two-tailed unpaired *t*-test was used: * *p* < 0.05, ** *p* < 0.01 vs. control. Abbreviations: SL, sialyllactose; BW, body weight.

Exercise performance and grip strength were assessed to evaluate the effects of 6′-SL on muscle functions in mice. For exercise performance, mice were orally administered 100 mg/kg of 6′-SL for 10 weeks and subjected to an exhaustive treadmill exercise test where the maximum running distance, time to fatigue, and work performed were measured. Administration of 6′-SL led to significant increases in distance (control: 560.6 ± 76.1 m; 6′-SL: 674 ± 87.1 m), time (control: 33.5 ± 2.7 min; 6′-SL: 37.1 ± 2.6 min), and work (control: 18,502.2 ± 2400.2 m × kg; 6′-SL: 23,140.7 ± 2929.4 m × kg) compared with the values observed in the control group (Figure 1A).

Grip strength was measured at weeks 3 and 10 of 6′-SL administration, and the rate of increase in grip strength between weeks 3 and 10 was calculated. The administration of 6′-SL led to a greater increase in grip strength than that observed in the control group (control: 96.3% ± 6.2%; 6′-SL: 108.6% ± 12%) (Figure 1B).

### 3.2. 6′-SL Increases the Volume and Size of GAS Muscles

**Figure 2:**
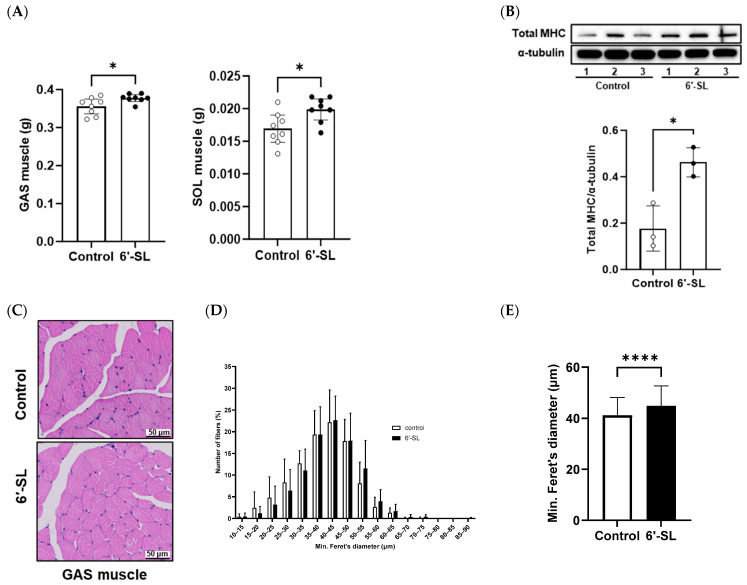
Effect of 6′-SL on muscle mass and fiber formation in GAS and SOL muscles. (**A**) Absolute tissue weight (gastrocnemius (GAS) and soleus muscles) was measured at sacrifice. (**B**) Protein expression levels of the total myosin heavy chain of GAS muscle were measured using Western blotting. The relative band intensities of each protein were normalized to those of α-tubulin, and the quantified data are shown in the bar graphs. (**C**) Images of hematoxylin- and eosin-stained cross-sections of the GAS muscle; scale bar: 50 μm. (**D**) Percentage muscle fiber distribution based on min. Feret’s diameter of muscle fiber size of the GAS muscle. The number of muscle fibers measured in the control and 6′-SL groups was 1535 and 1697, respectively. (**E**) Mean of min. Feret’s diameter muscle fiber size of the GAS muscle. The number of muscle fibers measured in the control and 6′-SL groups was 591 and 633, respectively. Data are presented as mean ± standard deviation values for each group (*n* = 8 for (**A**), *n* = 3 for (**B**–**E**)). White dots mean control groups and black dots mean 6′-SL groups. A two-tailed unpaired *t*-test was used: * *p* < 0.05, **** *p* < 0.0001 vs. control. Abbreviations: SL, sialyllactose; GAS, gastrocnemius; SOL, soleus; MHC, myosin heavy chain.

Muscle weights and fiber diameter were measured to examine the effects of 6′-SL on muscle phenotype characteristics. The administration of 6′-SL increased the absolute weights of the GAS and SOL muscles (Figure 2A), which are key muscles of the calf used for walking, running, and jumping. To support the observed increase in muscle weights, we measured body weight, fat mass, and various bone mass parameters (BMC, BMD, bone area, and bone volume) (Table 1). An increase in body weight without corresponding fat and bone mass gain was observed, partly suggesting an increase in muscle mass. Additionally, 6′-SL administration increased the protein expression of total MHC, a late-stage differentiation marker (Figure 2B) [24]. H&E staining of the GAS muscle revealed larger muscle fiber diameters in the 6′-SL administration group than in the control group. This finding was confirmed by quantifying fiber diameter size using a percentage distribution curve, which showed a trend toward larger sizes, and by measuring the average minimum Feret’s diameter, which was significantly increased in the 6′-SL administration group (Figure 2C–E).

## 4. Discussion

This study demonstrated that 6′-SL, an HMO, affected exercise performance by altering muscle mass, fiber size, and MHC protein expression in C57BL/6J mice. Exercise performance is influenced by intrinsic factors, such as genetic constitution and athletic ability, as well as extrinsic factors, such as exercise training and proper nutrition [25,26].

Muscle mass plays a key role in exercise performance, influencing many aspects of strength and athletic ability [27]. Increased muscle mass improves endurance by enabling greater force exertion and improved storage of glycogen, the main fuel required for prolonged exercise [28]. Various nutritional supplements have been reported to increase muscle mass, strength, and exercise performance [29]. For example, DHEA, a precursor of sex steroid hormones, promotes protein synthesis and anabolism, resulting in increased muscle mass and strength [30]. Beta-methyl-hydroxy-beta-methylbutyrate has demonstrated efficacy in preserving muscle mass and strength in older individuals and promoting skeletal muscle hypertrophy in bodybuilders and strength/power athletes [31,32]. Creatine has also been reported to positively affect several aspects of exercise performance, including muscle mass and strength, glycogen synthesis, and aerobic capacity [33]. Additionally, amino acids, including branched-chain amino acids, glutamine, aspartates, and arginine, as well as protein supplements, such as whey protein and colostrum, have demonstrated efficacy in improving physical performance [34]. Despite the availability of numerous dietary supplements, many uncertainties persist regarding their safety and effectiveness.

MHC is crucial for muscle function by influencing muscle contraction, energy consumption, and structure [35]. Additionally, MHC is a marker protein commonly used to assess muscle fiber lengths, muscle mass, and strength [36]. According to previous studies, various materials such as α-lipoic acid, creatine, dihydromyricetin, ursolic acid, and leucine have been identified to increase MHC expression and improve muscle function. α-lipoic acid, a potent biological antioxidant, has been reported to promote MHC gene expression and maintain muscle mass in Otsuka Long–Evans Tokushima Fatty rats and reduce muscle degradation, promote muscle regeneration, and consequently maintain muscle mass in rats with type 2 diabetes mellitus [36]. Creatine supplementation increased muscle strength and size through increased MHC mRNA and protein levels [37]. Dihydromyricetin has been shown to upregulate MHC I expression through the AMPK signaling pathway, enhancing muscle performance [38]. Moreover, a previous study showed that ursolic acid and leucine significantly induced MHC protein expression and promoted C2C12 muscle cell differentiation [39].

In this study, 6′-SL increased muscle weight (GAS and SOL), GAS muscle fiber size, and MHC protein expression in the GAS muscle. These results suggest that 6′-SL could enhance endurance exercise performance, including exhaustion time, total distance, and work output, by increasing muscle mass and strength in C57BL/6J mice.

Additionally, the safety of 6′-SL has been previously reported in piglets, healthy adults, and infant formulas [40,41,42].

Proposed mechanisms for improving muscle health include enhanced anti-inflammatory and antioxidant properties, increased expression of the mammalian target of the rapamycin (mTOR) signaling pathway, reduced protein degradation, and improved mitochondrial function [43,44]. Whey protein has been reported to induce the mTOR pathway in resistance-exercising young men [45,46]. Ginseng, which has antioxidant and anti-inflammatory properties, improves muscle regeneration post-exercise in healthy adults [47]. Branched-chain amino acids and vitamin D supplementation improved mitochondrial function and enhanced strength and performance in atrophic muscle [48]. Notably, the precise mechanism by which 6′-SL enhances exercise performance was not determined in this study. However, 6′-SL has been demonstrated to inhibit lipopolysaccharide-induced inflammatory symptoms in intestinally inflamed suckling mice [49]. Furthermore, in *Caenorhabditis elegans*, 6′-SL improved endurance exercise performance by increasing glycogenolysis and affecting mitochondrial function [50]. Based on these previous reports, we intend to conduct more comprehensive studies to elucidate the mechanisms by which 6′-SL affects muscle phenotype and functions.

Previous studies on the pathology of GNE myopathy have shown that 6′-SL has ameliorative effects in both mice and humans [19,20]. Based on these reports, 6′-SL may have clinical relevance for individuals in normal conditions, such as athletes and sports enthusiasts.

## 5. Conclusions

In conclusion, this study demonstrated that 6′-SL increased muscle mass and strength, thereby enhancing exercise performance in young C57BL/6J mice. These findings suggest that 6′-SL is both beneficial and safe for enhancing exercise performance. To develop 6′-SL as a dietary supplement to improve human exercise performance, testing in clinical settings including subjects such as athletes and sports enthusiasts will be required.

## Figures and Tables

**Table 1:** Data of dual-energy X-ray absorptiometry measurements.

	Control (*n* = 8)	6′-SL (*n* = 8)	*p*-Value
Body weight (g)	34.15 ± 1.40	35.92 ± 1.00 *	0.0164
Fat mass (g)	6.67 ± 1.13	6.83 ± 1.10	0.7987
Fat mass (%)	19.97 ± 3.24	19.43 ± 3.08	0.7542
BMC (g)	0.75 ± 0.05	0.78 ± 0.08	0.4361
BMD (g/cm^2^)	0.07 ± 0.00	0.07 ± 0.00	0.9153
Bone area (cm^2^)	11.33 ± 0.40	11.72 ± 0.71	0.2328
Bone volume (cm^2^)	0.45 ± 0.03	0.47 ± 0.05	0.4358

The levels of body weight, fat mass, bone mineral content, bone mineral density, bone area, and bone volume were evaluated. Data are presented as mean ± standard deviation values for each group (*n* = 8). A two-tailed unpaired *t*-test was used: * *p* < 0.05 vs. control. Abbreviations: SL, sialyllactose; BMC, bone mineral content; BMD, bone mineral density.

## Data Availability

The data presented in this study are available upon request from the corresponding author.
